# Supplementary figures and images for: Succession of physiological stages hallmarks the transcriptomic response of the fungus Aspergillus niger to lignocellulose
Source: Biotechnol Biofuels. 2020 Apr 13;13:69. doi: 10.1186/s13068-020-01702-2 (PMC7155255; doi:10.1186/s13068-020-01702-2)

FigS1

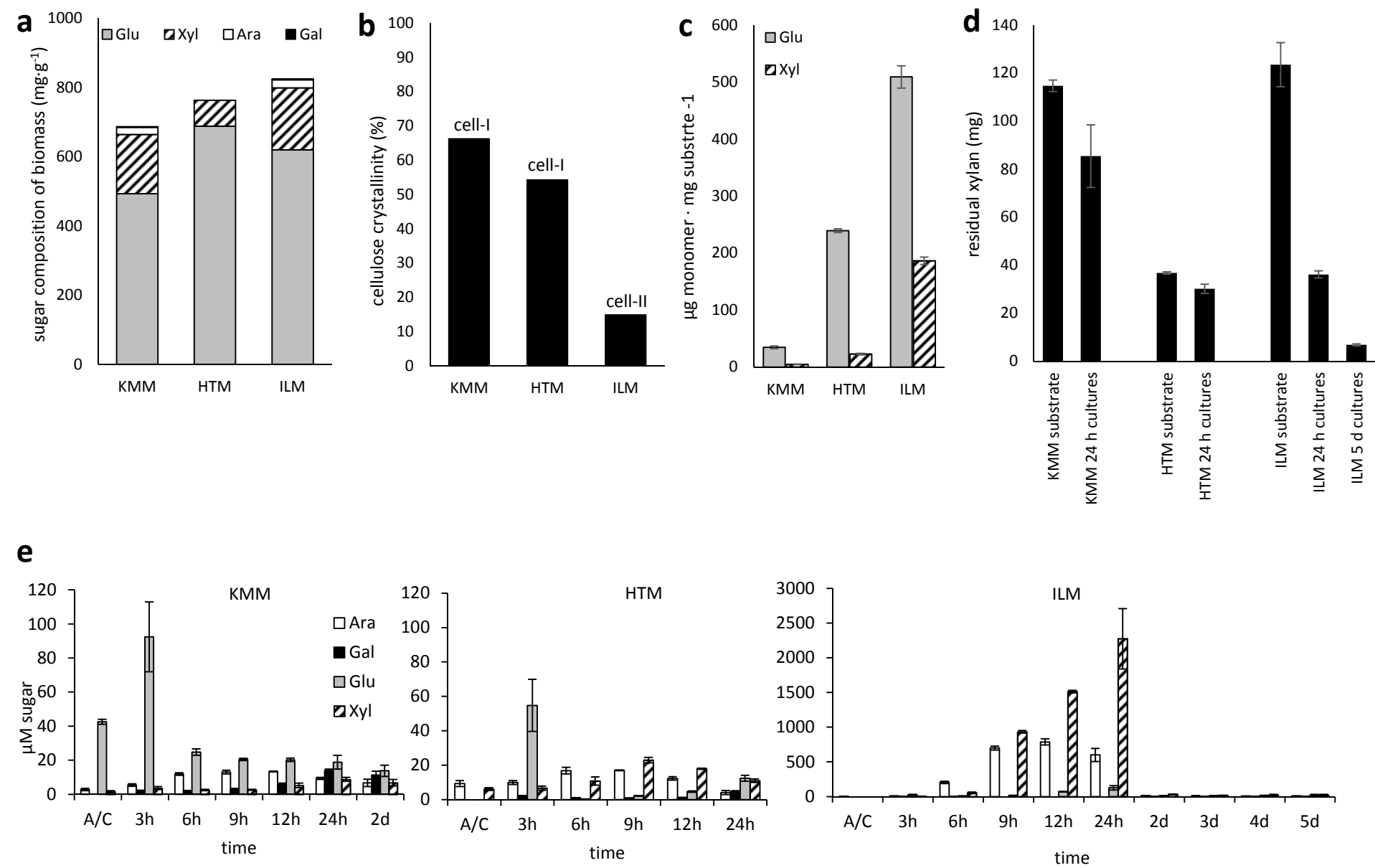

Supplement: Supplementary file 1 — Additional file 1: Figure S1. Characterisation of untreated and pretreated Miscanthus substrates. a Carbohydrate monomer composition of substrates, b substrate crystallinity with the predominant type of cellulose indicated c saccharification of substrates using commercial enzymes given as mean (n = 3) ± SE of released glucose and xylose after 72 h, d xylan in initial substrates and residual xylan from the solids recovered from the fungal cultures after 24 h and 5 d (where available), given as mean (n = 3) ± SE of biological replicate flasks or for the initial substrates as the mean ± SE of 2 technical hydrolysis replicates, e monosaccharide concentration in the fungal culture filtrates over time, given as mean (n = 3) ± SE with A/C only = autoclaved substrate only. [file 13068_2020_1702_MOESM1_ESM.pdf]

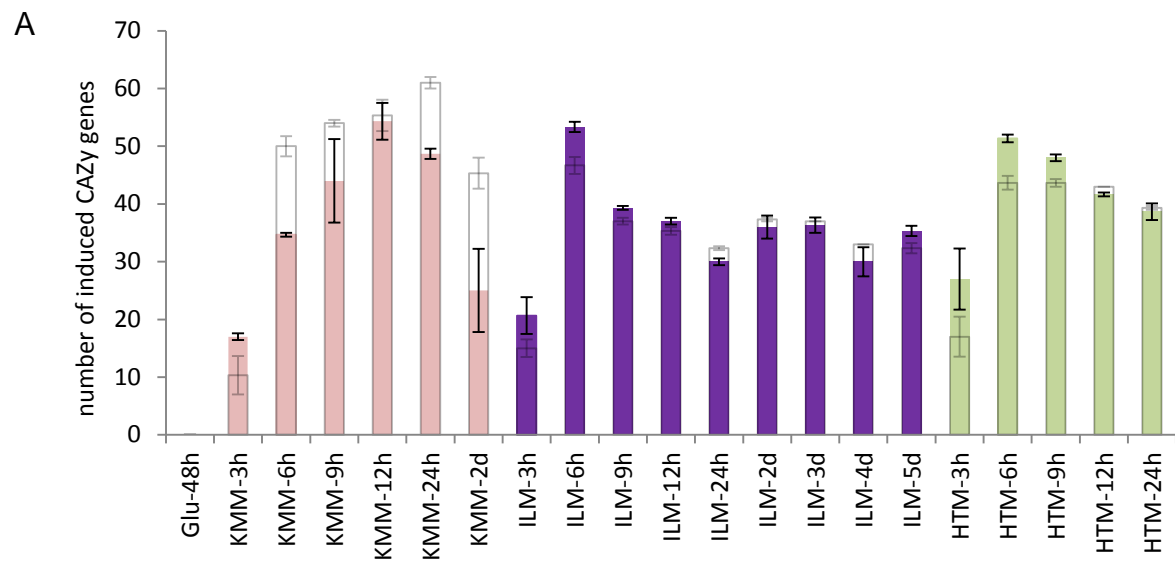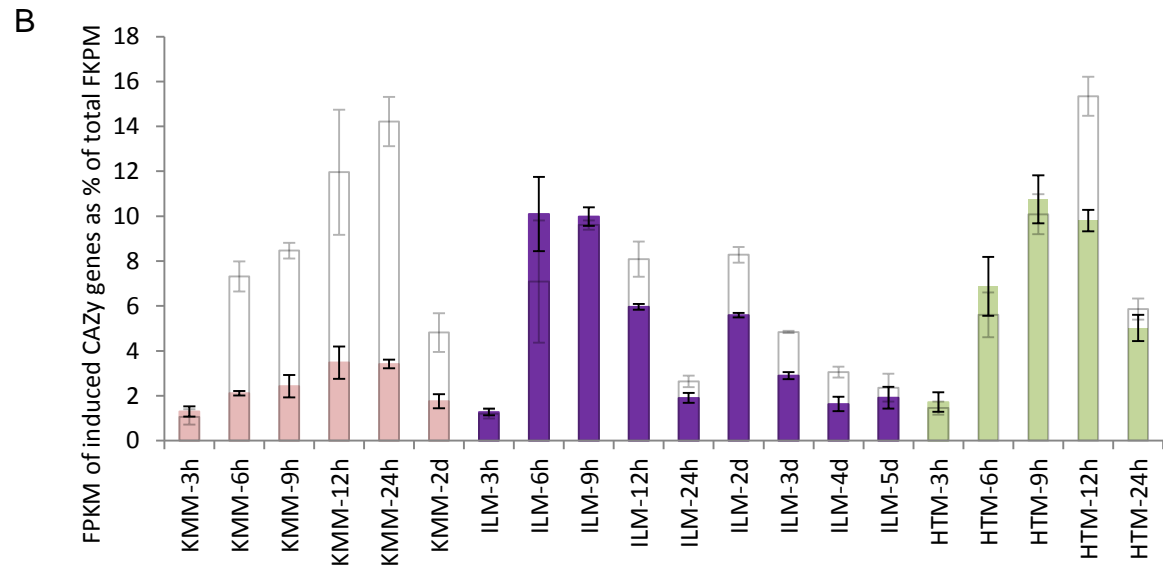

Supplement: Supplementary file 5 — Additional file 5: Figure S2. Expression of CAZyme encoding genes over Miscanthus time courses. a The total number of genes encoding plant-polysaccharide active CAZymes and that are significantly induced in cultures with untreated and pretreated Miscanthus as compared to the Glu 48 h control (DESeq padj < 0.05, FPKM of ≥ 50 on lignocellulose and log2 FC of ≥ 3). Values are mean ± standard errors (n = 3). b The proportion of transcripts from these CAZyme genes as percentage of total FPKMs. Error bars represent standard errors (n = 3). Colors indicate different culture condition groups and values in light grey are a comparison to the same data when using wheat straw as feedstock [31]. [file 13068_2020_1702_MOESM5_ESM.pdf]

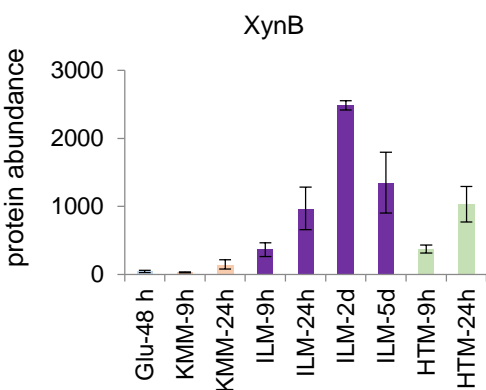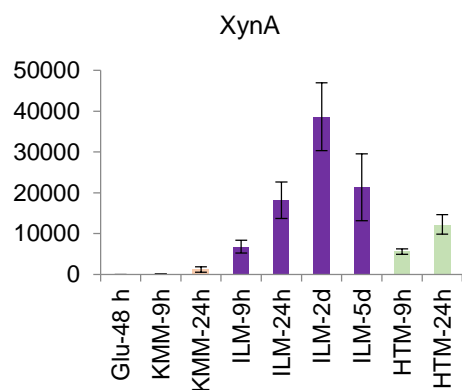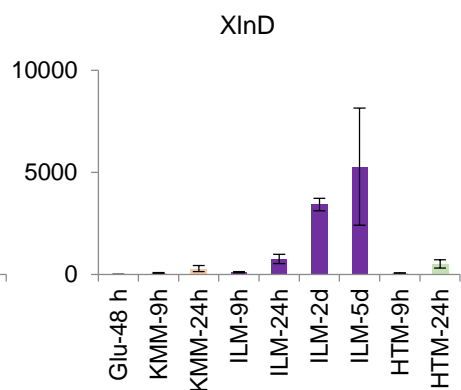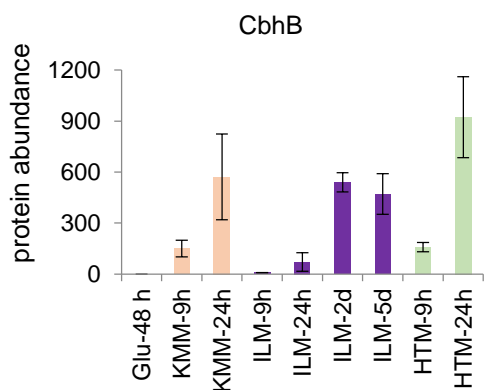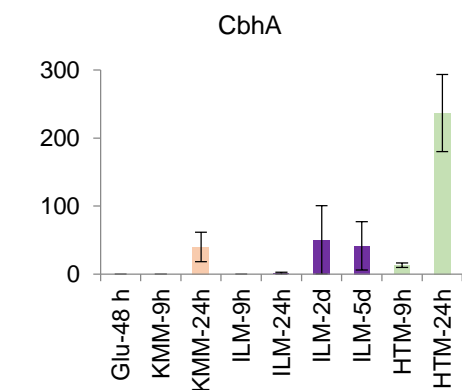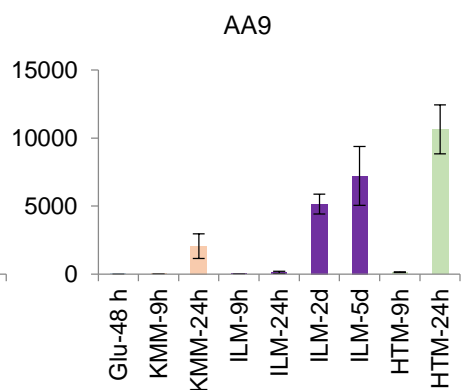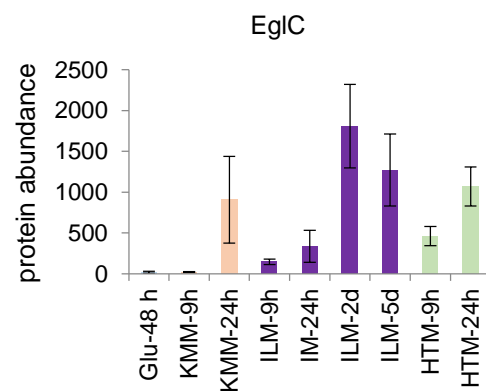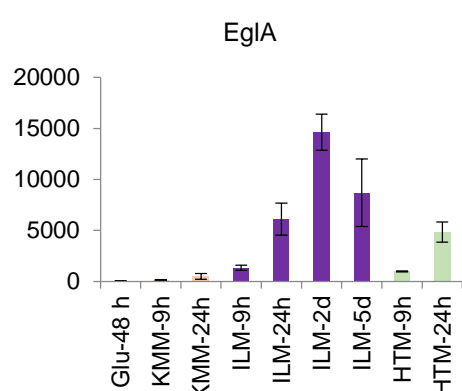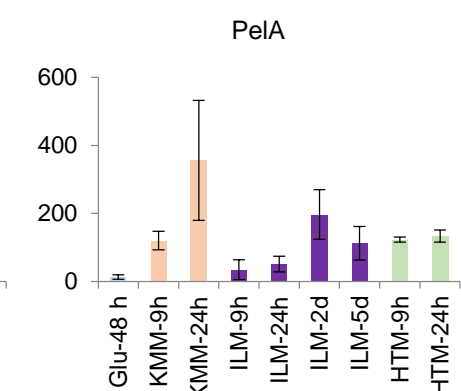

XynB

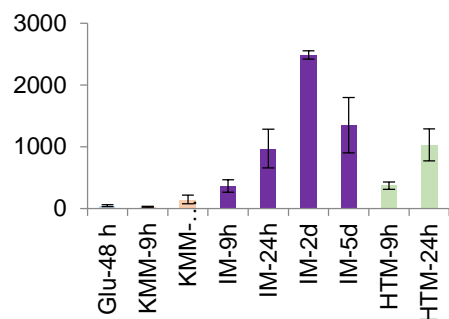

XynA

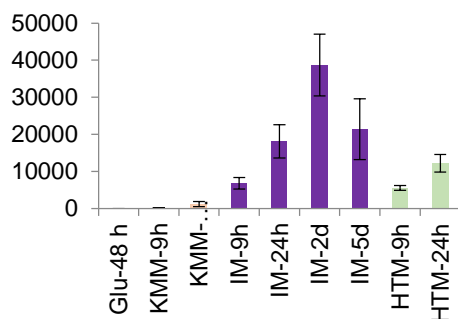

abfB

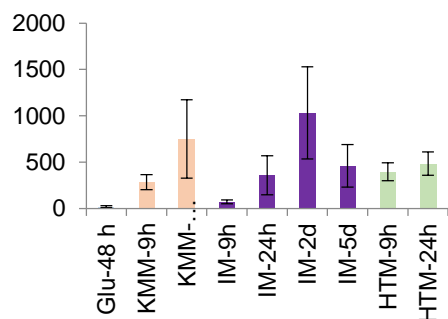

XlnD

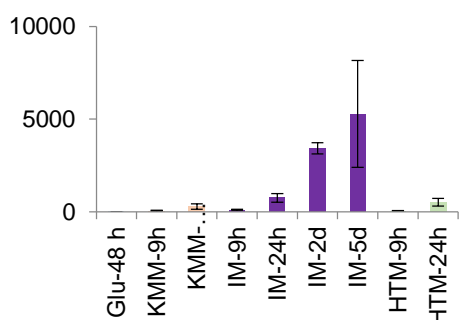

EglC xyloglucanase

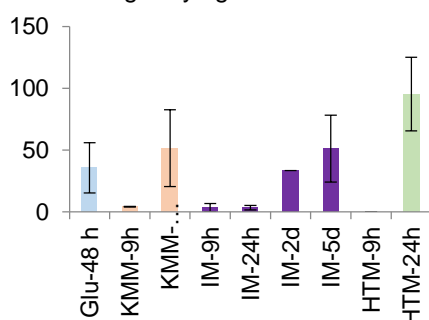

FaeA

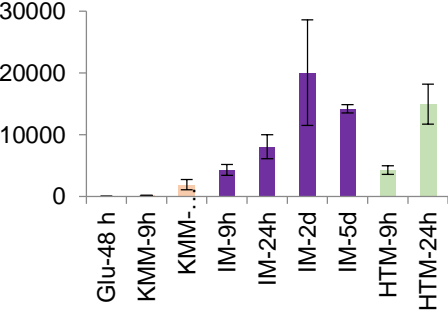

PelA

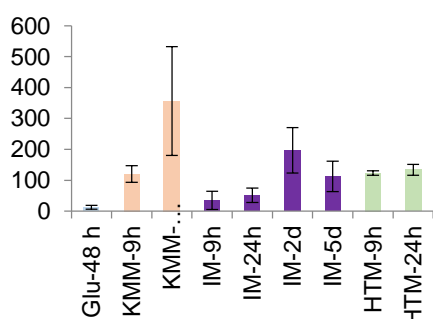

AbfA

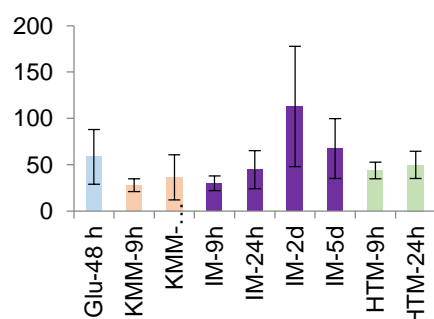

CbhB

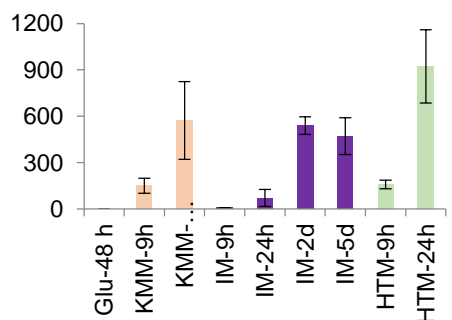

EglA

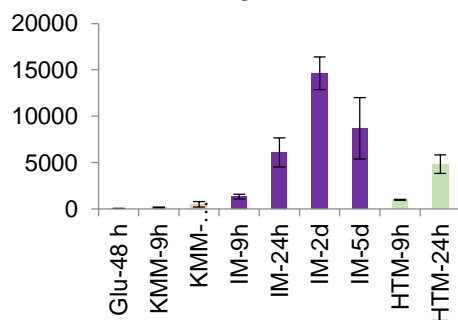

CbhA

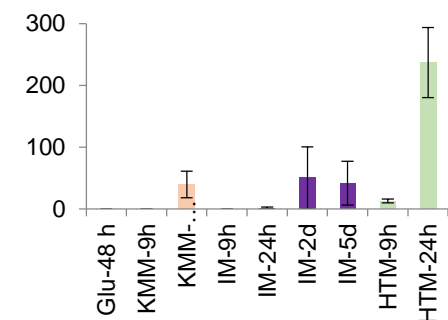

An03g01050

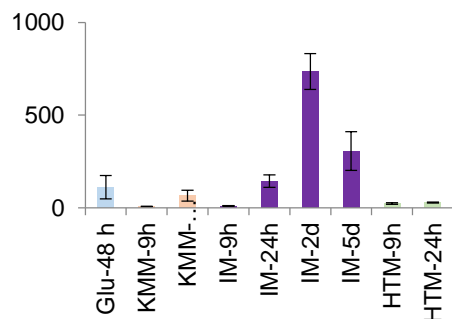

EglC cellulase

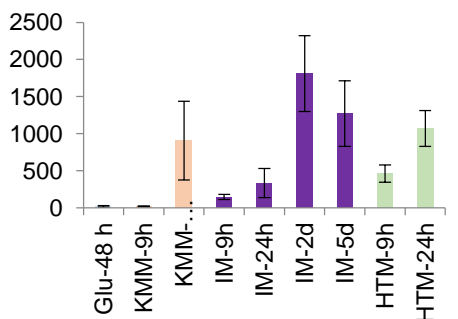

AA9

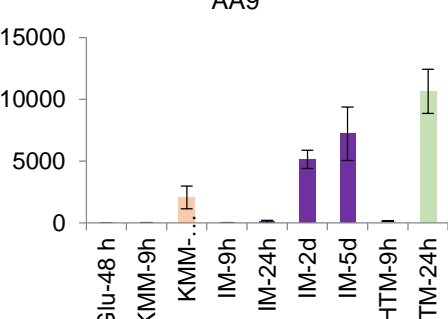

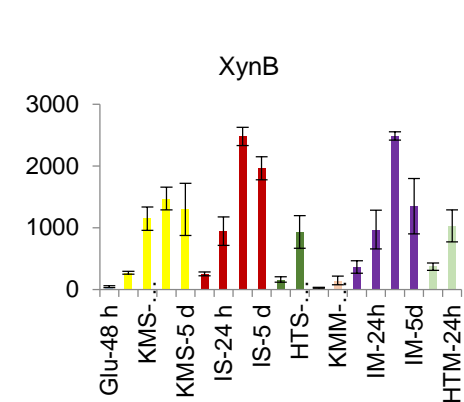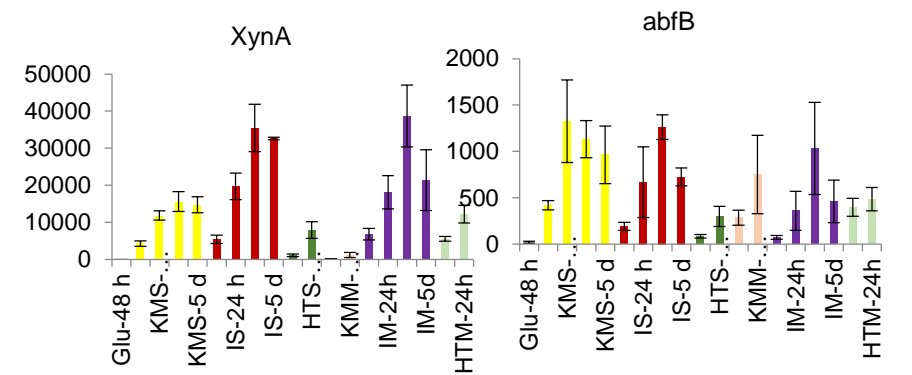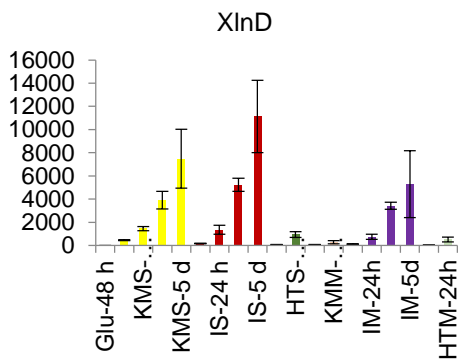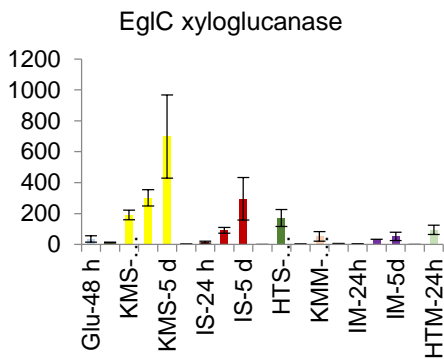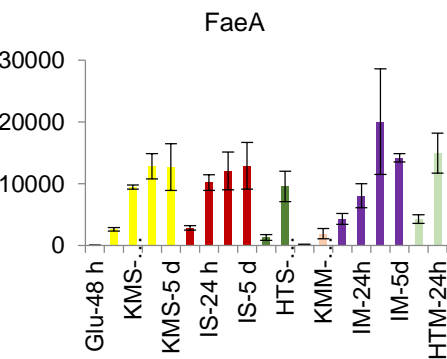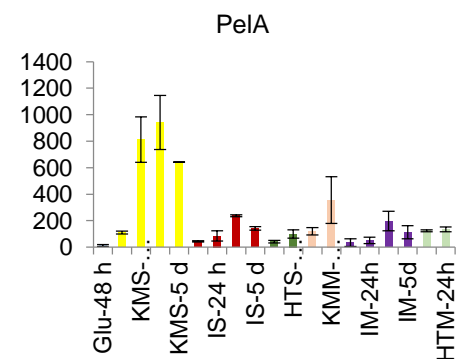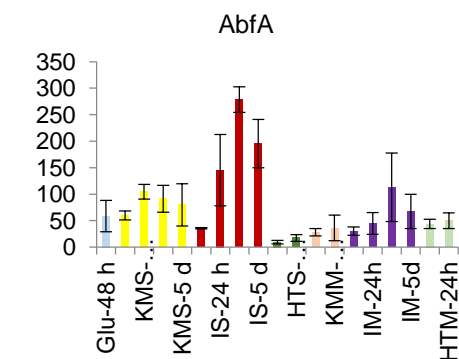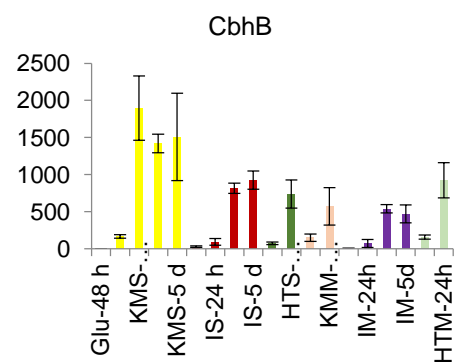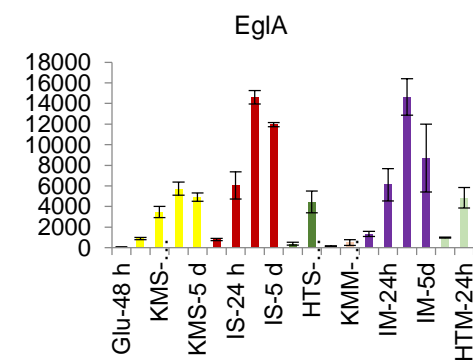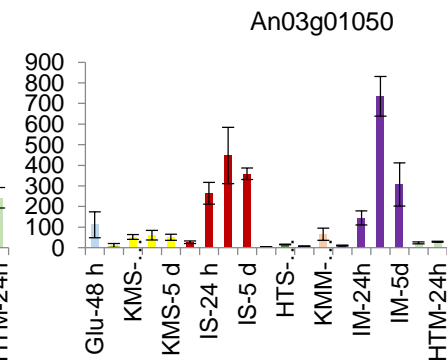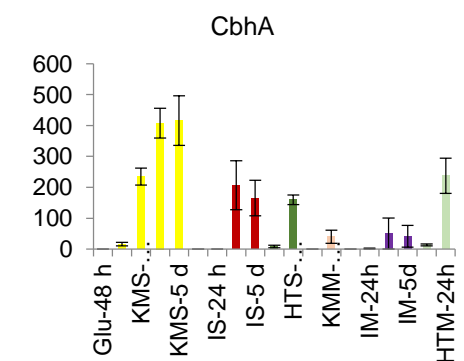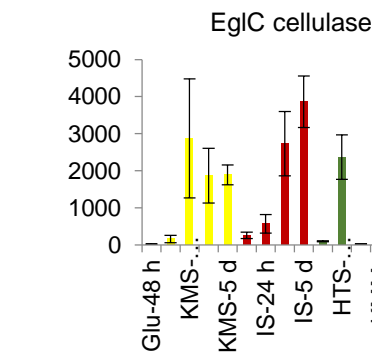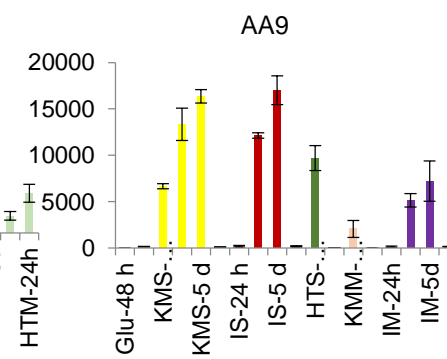

Supplement: Supplementary file 8 — Additional file 8: Figure S3. Targeted proteomics of selected proteins over the Miscanthus time courses. Relative abundance of the indicated enzymes are given as peak area corrected for total protein amount in culture filtrate. Values are given as mean ± st. error (n = 3) and gene identifiers corresponding to the proteins are given in Additional file 7: Table S5. [file 13068_2020_1702_MOESM8_ESM.pdf]
